# Daily exposure to formaldehyde and acetaldehyde and potential health risk associated with use of high and low nicotine e-liquid concentrations

**DOI:** 10.1038/s41598-020-63292-1

**Published:** 2020-04-16

**Authors:** Leon Kosmider, Sharon Cox, Marzena Zaciera, Jolanta Kurek, Maciej L. Goniewicz, Hayden McRobbie, Catherine Kimber, Lynne Dawkins

**Affiliations:** 1 Department of General and Inorganic Chemistry, Medical University of Silesia, Katowice FOPS in Sosnowiec, Jagiellonska 4, 41-200 Sosnowiec, Poland; 2 Centre for Addictive Behaviours Research, School of Applied Sciences, London South Bank University, SE1 0AA London, UK; 3 Department of Chemical Hazard and Genetic Toxicology, Institute of Occupational Medicine and Environmental Health, 41-200 Sosnowiec, Poland; 4 Roswell Park Cancer Institute, Department of Health Behavior, Buffalo, NY 14263 USA; 5 Queen Mary University of London, Wolfson Institute of Preventive Medicine, Barts and The London School of Medicine and Dentistry, E1 4NS London, UK

**Keywords:** Cancer, Disease prevention, Medical research

## Abstract

Recent evidence suggests that e-cigarette users tend to change their puffing behaviors when using e-liquids with reduced nicotine concentrations by taking longer and more frequent puffs. Using puffing regimens modelled on puffing topography data from 19 experienced e-cigarette users who switched between 18 and 6 mg/mL e-liquids with and without power adjustments, differences in daily exposure to carbonyl compounds and estimated changes in cancer risk were assessed by production of aerosols generated using a smoking machine and analyzed using gas and liquid chromatography. Significant differences across conditions were found for formaldehyde and acetaldehyde (p < 0.01). Switching from a higher to a lower nicotine concentration was associated with greater exposure regardless of whether power settings were fixed or adjustable which is likely due to increased liquid consumption under lower nicotine concentration settings. Daily exposure for formaldehyde and acetaldehyde was higher for 17/19 participants when using low (6 mg/mL) compared with high (18 mg/mL) nicotine e-liquid concentration when power was fixed. When power adjustments were permitted, formaldehyde and acetaldehyde levels were higher respectively for 16/19 and 14/19 participants with the use of 6 compared with 18 mg/mL nicotine e-liquid.

## Introduction

Although the long-term health effects of e-cigarette use remain unknown, published research indicates that e-cigarette aerosol contains far lower levels of toxins and carcinogens compared to tobacco smoke^1^. In a modelling study based on published data from chemical analyses of emissions from e-cigarette aerosols, Stephens concluded that e-cigarette emissions mostly have cancer potencies <1% of tobacco smoke, although there were some exceptions^2^. Similarly, biomarker studies have reported a more favorable toxicity profile in e-cigarette users compared with smokers^3–7^.

Nevertheless, a range of factors associated with both device characteristics and user behavior are likely to influence toxicity profile as well as nicotine exposure and quitting potential. Chemical emission studies using bench top tests/smoking machines have explored carbonyl compound emissions using different e-cigarette generations^3,8^, product characteristics^3,9,10^ flavors^11–14^ and e-liquid consumption^15^. These laboratory studies differ significantly in the study design and also levels of carbonyl yields^16,17^, which vary from trace levels to levels higher than in tobacco smoke^12,18^. Similarly, differences in nicotine levels in aerosol have been reported depending on type of device^19–23^, product characteristics^24–26^, and puffing topography^11,24,27^. Although non-human laboratory emission studies can be informative and cost-effective, the device set-up and puffing regimens used may not reflect real-world usage patterns. Thus, it has been suggested that e-cigarettes should be tested for safety under realistic conditions^28^ in order to better understand the long-term health effects of product use.

We have previously shown, in a study of e-cigarette users vaping in the laboratory^29^, that reduced nicotine concentration (6 mg/mL compared with 24 mg/mL) used within the same device results in compensatory puffing (longer and more frequent puffs), consequently increasing carbonyl mouth exposure to the users^30^. Nevertheless, vaping behavior in the lab, even under *ad libitum* conditions may not accurately reflect everyday ‘real-life’ usage, particularly when power can be adjusted as with newer generation devices. In a more recent companion study to the current paper^31^, we characterized e-cigarette use patterns (daily puff number, puff duration and inter-puff interval [IPI]) and changes to power (where permitted) outside the lab, across four conditions (each condition lasting a week): (i) low nicotine/fixed power); (ii) low nicotine/adjustable power; (iii) high nicotine/fixed power); iv) high nicotine/adjustable power (where low = 6 mg/mL and high = 18 mg/mL) Use of the lower nicotine concentration e-liquid was again associated with more intensive puffing patterns (higher number and duration of puffs) especially when power settings could not be adjusted. These studies clearly illustrate that when nicotine is reduced, vapers are likely to engage in compensatory behavior by increasing the power on their devices and/or puffing more intensively. Both power^8,15^ and puffing topography^30^ may affect toxicant emissions, but the extent to which such compensatory behavior affects daily toxicant exposure and subsequent health risk is unknown.

The current study aimed to estimate daily carbonyl exposure, change in cancer risk potency, aerosol yield and nicotine exposure associated with the puffing regimens used in our published human participant study^31^. We used a smoking machine in the laboratory to mimic vapers’ puffing patterns under the different nicotine e-liquid concentration and power conditions used in our previous study^31^. Toxicant yields per puff were then scaled up by the number of puffs taken per day by each participant in each condition to estimate daily toxicant exposure. We also performed a comparison of cancer risk potencies using the puffing regimens associated with use of both the high (18 mg/mL) and low (6 mg/mL) nicotine concentration e-liquids to determine whether compensatory behavior increased cancer risk potency. The main compounds of interest were carbonyl compounds (mainly formaldehyde and acetaldehyde), as the main carcinogens in e-cigarette aerosols^2^ and nicotine, because of its addictive potential. We hypothesized that the compensatory puffing patterns associated with using 6 (compared with 18) mg/mL nicotine e-liquid, particularly under adjustable power settings, would be associated with higher aerosol yield, higher formaldehyde and acetaldehyde emissions, and higher cancer risk potencies.

## Results

### Daily aerosol yield and nicotine exposure

Estimated daily aerosol yield and nicotine exposure were both calculated by multiplying the yield per puff by the mean daily number of puffs taken by each participant in each condition.

**Figure 1:**
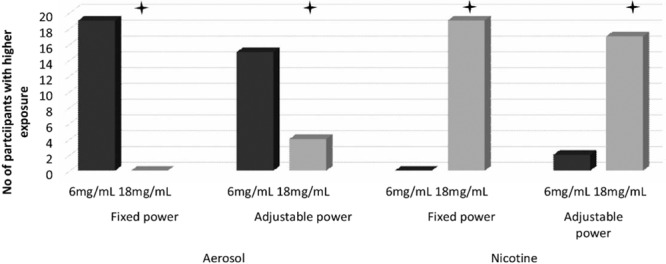
Number of participants with higher daily aerosol and nicotine mouth exposure with 6 vs. 18 mg/mL e-liquid under each power condition.

**Figure 2:**
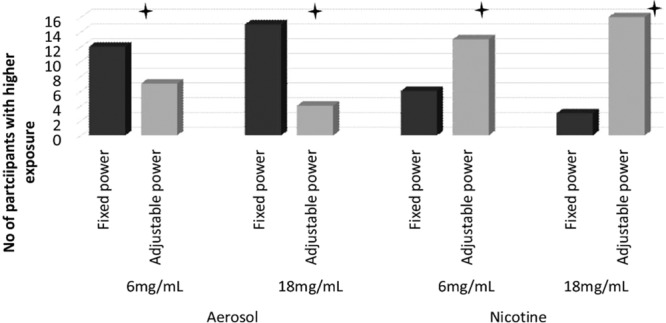
Number of participants with higher daily aerosol and nicotine mouth exposure with fixed vs. adjustable power settings under each e-liquid nicotine strength.

Table 1 presents the median (IQR) and mean (SD) values for the two nicotine strengths under fixed (F) and adjustable (A) voltage settings for daily aerosol yield and nicotine exposure whilst Figs. 1 and 2 illustrates the number of participants with increased or decreased exposure.

**Table 1:** Daily exposure to aerosol, nicotine, formaldehyde, acetaldehyde and formaldehyde & acetaldehyde per gram of aerosol yield.

Voltage	Fixed (F)						Adjustable (A)						Post hoc sig (<0.05)
Nicotine concentration (mg/mL)	6			18			6			18			
	N%	mean ± SD	Median (Q1-Q3)	N%	mean ± SD	Median (Q1-Q3)	N%	mean ± SD	Median (Q1-Q3)	N%	mean ± SD	Median (Q1-Q3)	
aerosol yield [g/day]	100	2.98 ± 1.96	2.26 (1.46–4.22)	100	1.67 ± 1.14	1.38 (0.7–3.01)	100	3.42 ± 2.35	2.64 (1.71–4.85)	100	2.00 ± 1.20	1.77 (1.22–2.57)	6 F > 18 F; 6 A > 18 A;6 F > 6 A; 18 F > 18 A
nicotine [mg/day]	100	13.22 ± 8.93	10.26 (7.43–16.94)	100	22.69 ± 15.16	17.88 (10.23–40.04)	100	15.23 ± 10.49	13.53 (8.88–19.37)	100	27.87 ± 16.86	23.96 (17.73–37.76)	18 F > 6 F;18 A > 6 A; 6 A > 6 F;18 A > 18 F
formaldehyde [µg/day]	100	141.08 ± 389.44	26.83 (11.99–56.11)	100	15.81 ± 10.68	13.69 (6.95–27.99)	100	229.54 ± 451.41	25.63 (15.82–70.74)	100	118.46 ± 291.51	20.23 (10.1–45.57)	6 F > 18 F;6 A > 18 A 18 A > 18 F
acetaldehyde [µg/day]	100	47.89 ± 74.11	19.91 (4.68–66.47)	100	19.09 ± 22.72	8.18 (3.42–34.03)	100	79.86 ± 118.23	20.17 (5.61–83.00)	100	47.86 ± 74.59	15.54 (4.87–51.26)	6F > 18F; 6A >18A;18A > 18F
Formaldehyde/g of aerosol [µg/g]	100	26.87 ± 54.69	9.31 (7.24–16.15)	100	9.82 ± 2.07	10.27 (8.83–11.40	100	47.71 ± 89.60	10.58 (8.58–16.26)	100	40.56 ± 85.18	11.34 (8.51–20.23)	ns
Acetaldehyde/g of aerosol [µg/g]	100	20.29 ± 30.36	5.21 (2.42–38.69)	100	19.16 ± 31.31	4.33 (2.59–20.62)	100	28.13 ± 39.06	5.15 (2.50–48.54)	100	25.66 ± 35.41	8.78 (2.95–35.95)	ns

Results were analysed using non-parametric Friedman tests on medians with post-hoc Wilcoxon signed rank tests to compare differences between conditions. Q1 and Q3 –first and third quartile

In the 6 mg/mL nicotine condition, the median g/day e-liquid consumed was higher under adjustable (A) compared with fixed (F) voltage settings. In the 18 mg/mL nicotine condition, consumption was also higher under F and A voltage settings. Friedman analysis showed statistically significant differences between conditions (6F, 18F, 6A, 18A: p < 0.01, N = 19, DF = 3, *Χ*^2^ = 33.4).

Wilcoxon Signed Ranks Tests revealed a significant difference between 6 mg/mL and 18 mg/mL within both F and A settings. Expressed as frequencies, 19/19 users increased their e-liquid consumption when using 6 compared with 18 mg/mL nicotine concentration e-liquids when voltage settings were fixed and 15/19 increased their consumption in the 6 vs 18 mg/mL condition when settings were adjustable (6F vs 18F and 6A vs. 18A, p < 0.01).

There was also a statistically significant difference in e-liquid consumption between F and A settings within the same nicotine strength e-liquid (for both 6 and 18 mg/mL p < 0.05); 12/19 users had higher daily e-liquid consumption when comparing 6F to 6A and 15/19 when comparing 18F to 18A.

Similar differences were found for daily nicotine exposure (Friedman test: p < 0.01, N = 19, DF = 3, *Χ*^2^ = 41.8). Average values respectively for fixed and adjustable settings are shown in Table 1.

Wilcoxon Signed Ranks Test revealed a significant difference between 6 mg/mL and 18 mg/mL within both the F and A settings. 17/19 users had higher nicotine mouth exposure when using 18 mg/mL e-liquid compared to 6 mg/mL e-liquid under adjustable (A) power settings (p < 0.01). When power settings were fixed (F), all participants had higher daily mouth nicotine exposure in the 18 mg/mL condition (p < 0.01).

There were also statistically significant differences between F and A settings within the same nicotine strength e-liquid. 13 and 16/19 participants were exposed to higher daily amount of nicotine inhaled under A compared to F settings for 6 (p = 0.04) and 18 mg/mL (p = 0.01) nicotine e-liquids respectively.

### Daily exposure to carbonyl compounds with carcinogenic potential

**Figure 3:**
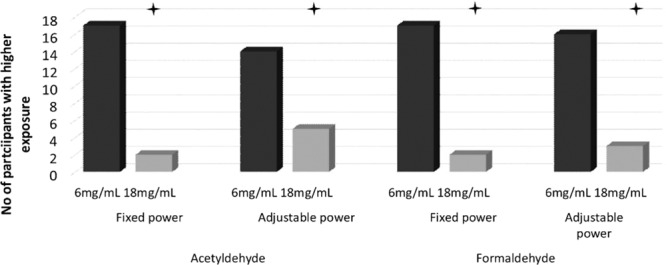
Number of participants with higher daily acetaldehyde and formaldehyde mouth exposure with 6 vs. 18 mg/mL e-liquid under each power condition.

**Figure 4:**
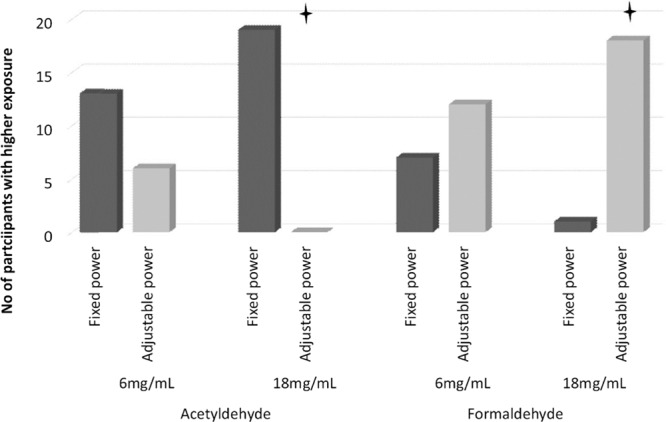
Number of participants with higher daily acetaldehyde and formaldehyde mouth exposure with fixed vs. adjustable power settings under each e-liquid nicotine strength.

The chromatographic method used in our study allowed us to measure 15 different carbonyl compounds in e-liquids and e-cigarette aerosol. However, the main focus of this paper is to compare health risk to the user, so a statistical analysis was performed only for formaldehyde and acetaldehyde. Estimated daily carbonyl exposure was calculated by multiplying the carbonyl yield per puff by the mean daily number of puffs taken by each participant in each condition. The median (IQR) and means (SD) for the analysed compounds can be found in Table 1 and all other non-analysed compounds quantified are shown in Table 2. Graph(s) depicting the number of participants who increased their formaldehyde and acetaldehyde exposure (as per our Friedman analysis) can be found in Figs. 3 and 4. In the analysis that follows, only median values are presented, as carbonyl yield characteristically adds skewness to the data.

**Table 2:** Daily exposure to carbonyl compounds not analysed in the main paper.

Voltage	Fixed						Adjustable					
Nicotine concentration (mg/mL)	6			18			6			18		
	N%	mean ± SD	Median (Q1-Q3)	N%	mean ± SD	Median (Q1-Q3)	N%	mean ± SD	Median (Q1-Q3)	N%	mean ± SD	Median (Q1-Q3)
acrolein [µg/day]	72	*103.5 ± 361.08	5.15 (1.06–34.52)	56	*6.05 ± 8.1	1.31 (0.68–10.59)	84	*101.6 ± 296.82	15.16 (2.03–53.45)	74	*27.31 ± 52.82	5.1 (1.13–25.14)
acetone [µg/day]	91	*13.84 ± 12.59	10.22 (4.04–25.15)	100	8.94 ± 7.53	6.21 (2.91–18.38)	95	*13.01 ± 11.92	9.09 (5.37–17.98)	88	*10.75 ± 10.02	8.49 (3.86–13.71)
propionaldehyde [µg/day]	88	*5.45 ± 15.01	1.54 (1.02–2.82)	70	*1.05 ± 0.9	0.85 (0.41–1.64)	89	*10.19 ± 20.15	2.21 (1.25–3.64)	77	*5.39 ± 10.45	1.61 (0.43–2.03)
crotonaldehyde [µg/day]	12	*1.01 ± 2.94	0.17 (0–0.52)	7	*0.46 ± 1.52	0 (0–0.32)	25	*1.48 ± 3.84	0.39 (0–0.89)	26	*0.62 ± 1.03	0.21 (0–0.64)
butyraldehyde [µg/day]	44	*0.83 ± 1.01	0.33 (0–1.89)	53	*0.88 ± 1.26	0.4 (0.13–1.45)	40	*1.25 ± 2.17	0.39 (0–1.73)	47	*0.57 ± 0.63	0.26 (0.11–1.22)
benzaldehyde [µg/day]	53	*3.84 ± 6.69	0.49 (0–8.2)	47	*1.56 ± 2.89	0.25 (0–1.27)	47	*1.84 ± 3.1	0.4 (0–2.79)	53	*1.43 ± 2.36	0.37 (0.2–1.15)
isovaleraldehyde [µg/day]	14	*0.15 ± 0.34	0 (0–0.3)	11	*0.19 ± 0.55	0 (0–0.21)	7	*0.18 ± 0.6	0 (0–0)	2	*0.03 ± 0.1	0 (0–0)
valeraldehyde [µg/day]	81	*11.33 ± 40.91	0.95 (0.39–3.17)	77	*0.88 ± 0.72	0.58 (0.32–1.31)	89	*13.95 ± 37.32	1.58 (0.51–4.2)	86	*3.15 ± 6.37	1.04 (0.47–2.06)
o-methylbenzaldehyde [µg/day]	89	*10.29 ± 7.79	8.24 (5.3–13.61)	81	*4.57 ± 4.28	2.99 (1.56–6.79)	95	*8.44 ± 6.04	8.04 (4.38–10.15)	93	*4 ± 1.84	3.57 (2.92–5.11)
m-methylbenzaldehyde [µg/day]	9	*0.55 ± 1.54	0 (0–0)	2	*0.13 ± 0.4	0 (0–0)	25	*1.03 ± 1.76	0 (0–2.37)	11	*0.48 ± 1.13	0 (0–0)
p-methylbenzaldehyde [µg/day]	100	45.47 ± 56.17	31.19 (13.73–55.37)	100	16.9 ± 16.12	10.28 (6.01–27.77)	100	42.57 ± 35.07	32.77 (17.3–55.35)	100	21.08 ± 13.51	18.52 (9.59–29.46)
hexanal [µg/day]	53	*36.87 ± 104.61	0.61 (0.38–24.45)	47	*16.66 ± 40.36	0.48 (0.36–4.1)	37	*24.14 ± 63.93	0.44 (0–20.05)	39	*7.74 ± 16.32	0.47 (0.2–5.66)
2,5-dimetylobenzaldehyde [µg/day]	5	*0.2 ± 0.43	0 (0–0)	4	*0.17 ± 0.33	0 (0–0)	5	*0.19 ± 0.42	0 (0–0)	0	*0.16 ± 0.33	0 (0–0)

N% percentage of samples above limit of quantification in the raw data; *mean value and standard deviation (SD) is only approximation, as artificial values were putted instead of BLQ and ND (see methodology); Q1 and Q3 –first and third quartile.

There was a significant difference across conditions in relation to formaldehyde (Friedman Test p < 0.01, N = 19, DF = 3, *Χ*^2^ = 29.4). The median of daily mouth exposure to formaldehyde for the 6 mg/mL nicotine e-liquid condition was similar across fixed versus adjustable (A) settings. For the 18 mg/mL condition, the median was higher under adjustable compared to fixed settings (see Table 1).

In terms of frequencies (shown in Fig. 3), daily mouth exposure for formaldehyde was higher for 17/19 participants under Fixed settings and for 16/19 under Adjustable settings when the 6 mg/mL nicotine e-liquid was used in comparison with the 18 mg/mL nicotine e-liquid (both p < 0.01). When comparing differences for Fixed vs. Adjustable within the same nicotine strength (shown in Fig. 4), 18/19 participants had higher daily mouth exposure under Adjustable vs. Fixed settings when using 18 mg/mL (p < 0.01). 12/19 participants had higher daily mouth exposure under Adjustable vs. the Fixed settings when using 6 mg/mL liquid (p > 0.05).

A similar pattern was observed for acetaldehyde, where median values were similar for F and A settings for the 6 mg/mL nicotine e-liquids condition, and lower for F and A settings for 18 mg nicotine e-liquid (see Table 1). Friedman Test showed significant differences between conditions (p < 0.01, N = 19, DF = 3, *Χ*^2^ = 27.8).

Wilcoxon Signed Rank Test revealed differences in acetaldehyde daily mouth exposure between 6F and 18F and 6A and 18A. 17/19 participants had higher values in the 6 vs. 18 mg/mL nicotine e-liquid condition when settings were fixed (p < 0.01) and 14/19 had higher values in the 6 vs 18 mg/mL nicotine e-liquid condition when settings were adjustable (p < 0.05; see Fig. 3). Comparisons between fixed versus adjustable settings showed that, for 18 mg/mL nicotine use, all participants had higher daily mouth exposure to acetaldehyde under Adjustable vs Fixed (p < 0.01). In the 6 mg/mL condition, 13/19 participants had higher daily mouth exposure when settings were Adjustable vs Fixed, this difference did not reach statistical significant (p > 0.05) (See Fig. 4)

To test for differences in daily carbonyls exposure derived by liquid consumption, carbonyl exposure per gram of aerosol yield was calculated and Friedman tests performed. The results for both formaldehyde/g and acetaldehyde/g of aerosol vaporized, Friedman tests were p = 0.15 and p = 0.19. These values are presented in the Table 1.

### Estimation of cancer risk associated with compensatory behaviors

Our simulation suggests that the Cancer Risk Index (CRI) for formaldehyde [median (1^st^ quartile – 3^rd^ quartile)] was 8.1 × 10^−6^ (3.6 × 10^−6^ – 1.7 × 10^−5^) when participants used a low nicotine concentration (6 mg/mL) without adjusting power (6F) and 4.1 × 10^−6^ (2.1 × 10^−6^ – 8.4 × 10^−6^) in the comparable power condition when using the higher nicotine concentration (18F). This represents a potential 1.98 fold increase in cancer risk associated with inhaling higher doses of formaldehyde with 6 mg/mL compared with 18 mg/mL e-liquid use. When power was adjustable, the CRI for 6 mg/mL nicotine concentration e-liquid (6A) was 7.7 × 10^−6^ (4.8 × 10^−6^ – 2.1 × 10^−5^) and for 18 mg/mL nicotine concentration e-liquid (18A), it was 6.1 × 10^−6^ (3.0 × 10^−6^ – 1.4 × 10^−5^) with a 1.26 fold increase for 6 compared with 18 mg/mL e-liquid nicotine use.

For acetaldehyde [median (1^st^ quartile – 3^rd^ quartile)], when using low nicotine concentrations without adjusting power (6F) the CRI was 2.7 × 10^−6^ (6.3 × 10^−7^ – 9.0 × 10^−6^). For 18F it was 1.1 × 10^−6^ (4.6 × 10^−7^ – 4.6 × 10^−6^) representing a potential 2.45 fold increase in cancer risk associated with acetaldehyde with the use of 6 vs. 18 mg/mL e-liquid. When users could adjust power, the CRI for low nicotine (6A) was 2.7 × 10^−6^ (7.6 × 10^−7^ – 1.1 × 10^−5^); and 2.1 × 10^−6^ (6.6 × 10^−7^ – 6.9 × 10^−6^) for high nicotine (18A) representing a 1.3 fold increase for 6 compared with 18 mg/mL e-liquid nicotine use.

**Figure 5:**
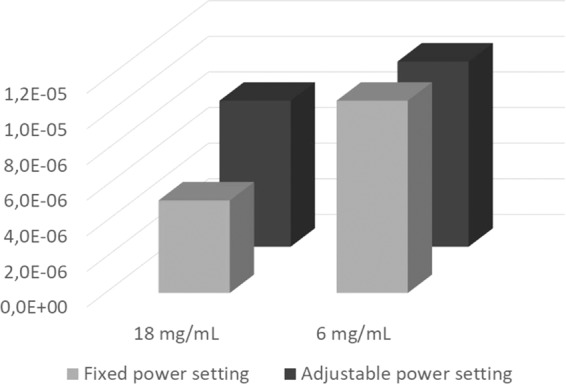
CRI comparison between different experiments settings.

When combining cancer potency risk from formaldehyde and acetaldehyde, our simulation suggests a 2.06 fold increase in cancer risk with fixed voltage (F) and a 1.27 fold increase with adjustable voltage (A) when switching from 18 mg/mL to 6 mg/mL e-liquids (see Fig. 5)

## Discussion

This study estimated exposure to carbonyl compounds from e-cigarette aerosol based on real-life topography data gathered over one week in a linked study previously published^31^. Here we used the same e-liquids and devices as Dawkins *et al*. and the mean puffing topography data from each participant under each experimental setting as used by participants. The main aim of this study was to assess any influence of changing usage patterns (compensatory puffing) of the same device under different nicotine concentration and voltage settings on daily exposure to carbonyls and cancer risk potencies. We also estimated the daily aerosol yield and nicotine exposure associated with the usage patterns under each condition.

Using the participant puffing patterns associated with use of the lower (6 mg/mL) nicotine concentration e-liquid from our previous study^31^, here we demonstrate that significantly more participants increased their daily aerosol yield exposure, formaldehyde exposure and acetaldehyde exposure in both the fixed (F) and adjustable (A) voltage settings. After applying methods used by Stephens^2^ to compare cancer potencies of e-cigarette aerosol, we found that depending on experimental settings, cancer potency factor can increase two to two and half times for users switching from 18 to 6 mg/mL nicotine e-liquids. It is important to note however, that these values are still lower than tobacco smoke for those compounds, as reported previously^2,32^.

In fact, CRI values (1.1 × 10^−6^ − 7.7 × 10^−6^) reported here approximate the results obtained by Stephens^2^ for e-cigarettes (9.5 × 10^−5^) and medical inhalers (8.9 × 10^−6^); and, the differences can be explained by different puffing behaviors and devices used in Stephens’ dataset and by the fact, that our team concentrated on carbonyl exposure only. When those values are compared to the CRI calculated by Stephens for tobacco cigarettes (2.4 × 10^−2^), the ratio to cigarettes smoked CRI (15 cigarettes per day) to our results was found to be 3116.9 to 21818.2 times higher. In other words, these findings suggest tobacco smoking has a carcinogenic potential of magnitude 3116.9 to 21818.2 times greater than e-cigarette use. Thus, our values represent only 95% of the total CRI as mentioned by Stephens^2^.

While there are some suggestions for an association between exposure to formaldehyde and increased incidence of lung and nasopharyngeal cancer, it is worth noting that the CRI methodology assumes that cancer risk is linear in the whole range of exposure. In fact, studies suggesting such associations may be limited due to possible exposure to other agents that may have contributed to causing cancers^33^. On the other hand, animal models reveal that cancer potential of formaldehyde might be not linear, especially in low concentrations. As showed by Monticello *et al*.^34^ concentration of formaldehyde of 2ppm (2.4 mg/m3) for 6 h a day for 5 days per week up to 24 months do not cause cancer in rats. Sharp increases in tumor incidence in the nasal cavity occurred only at concentrations greater than 6 ppm (7.2 mg/m3) formaldehyde. Those results are supported by newer analyses^35^. To compare those values to the values we found in our studies, conservative assumptions (respiratory minute volume-40L; assuming that participants where using e-cigarette for 16 h/day) were taken. Hypothetical exposure concentrations between the lowest Q_1_ and the highest Q_3_ values taken (6.95 µg/day for 18 mg F setting and 70.74 µg/day for 6 mg A setting), were within range from 0.0015 ppm (0.0018 mg/m3) to.015 ppm (0.018 mg/m3). These are much lower than the values which were proven to cause cancer in animal models (6 ppm). Similar analyses for acetaldehyde are difficult due to the lack of sufficient animal models assessing the carcinogenicity of low concentrations acetaldehyde by inhalation.

These findings are consistent with our previous work which, through the use of two different methods (analysis of aerosol generated in the lab and exposure to biomarkers of exposure in human urine), has indicated higher formaldehyde exposure when lower nicotine e-liquids are used^31^. Here, most participants showed an increase in daily exposure to all carcinogenic or potentially carcinogenic carbonyl compounds when the device was used with the lower nicotine concentration e-liquid. Our results suggest that aerosol yield and liquid consumption per day were the main determinants of the differences in daily formaldehyde and acetaldehyde exposure between conditions. This finding is in agreement with studies reporting that while aldehydes levels per puff are dependent on power settings, aldehydes levels per gram liquid consumption are independent of power settings when the devices are used in realistic conditions^15,18^. Still, a limitation of this particularly analysis is that there may not be enough statistical power to detect differences between conditions in aldehyde exposure per g of liquid consumption. Nevertheless, increasing nicotine delivery while keeping carbonyl emissions low should potentially reduce any health risk from vaping. This was demonstrated recently in a study of JUUL^36^ which delivers high nicotine but operates at low power by limiting its peak temperature. Nicotine-normalized formaldehyde and total carbonyl yields were lower respectively by six fold and 50 fold compared to combustible cigarettes and lower than other previously studied e-cigarettes.

However, analysis of carbonyl compounds in the liquid, did reveal some differences in formaldehyde and acetaldehyde content between different bottles of the same batch which reached up to 30%. Interestingly, the amount of those two compounds differed between the 6 mg/mL and 18 mg/mL nicotine concentration e-liquids used in the study (within the same brand of e-liquid), usually, although not consistently, higher amounts were found in the 6 mg/mL liquids. Although this might have had an influence on carbonyls yield, it should not influence the general trend, as the daily exposure to formaldehyde and acetaldehyde exceeded the yield of those compounds which would be found in the amount of e-liquids vaporized daily. This suggests that most of the formaldehyde and acetaldehyde exposure is a result of degradation of e-liquid components. Previous reports suggest that formaldehyde and acetaldehyde in aerosol might be related to the power of the device and the composition of the e-liquid^3,16,27,37–39^.

Some of the results were below the limit of detection and artificial value was used in those cases. This however, should not influence our results as non-parametric analyses was performed. In our analysis of cancer risk, only carbonyls were considered, as we did not quantify other carcinogenic compounds, so the results of relative cancer risk potential might be underestimated. However, according to Stephens, carbonyl compounds are responsible for more than 95% of cancer potency of e-cigarette aerosol^2^.

We also checked whether the actual nicotine content of the liquid corresponded to the labels on the bottles. In each case, we analyzed three bottles from the batch used in the study and nicotine content in the liquid differ substantially from bottle labels. For 11 of 14 samples (79%), the actual nicotine content was within 10% of the value on the labels. These results are largely consistent with the literature with the exception of a few previous reports^40–44^. Three samples, which did not meet targeted concentrations, had lower than declared nicotine levels; this may be explained by the degradation of the product over time. The variation between different bottles of the same e-liquid did not exceed 10% (relative standard deviation). Thus, we concluded, that the quality of e-liquids used in this study do not differ significantly from average e-liquids available on the market^44–46^.

This study is also the first attempt to describe daily mouth exposure to nicotine, aerosol yield and carbonyl compounds, based on puffing patterns and emissions from an e-cigarette. Daily nicotine exposure depended on nicotine concentration used and whether voltage was fixed or adjustable. Although using a lower nicotine level e-liquid, especially under fixed voltage conditions, was related to lower nicotine exposure in comparison with higher nicotine e-liquid, the results of our daily aerosol exposure suggest that e-cigarettes were used more intensively with lower nicotine concentration e-liquids, supporting our previous findings^29,30^. These results suggest that compensatory behaviors such as number of puffs, puff duration and device power are insufficient to inhale equal amount of nicotine after switching from 18 mg/mL to 6 mg/mL using the same device. Thus, our findings suggest switching to lower nicotine concentrations might increase liquid consumption (through compensatory puffing) result in higher carbonyl exposure, and a decrease in nicotine exposure. Thus, a regulatory framework which restrict nicotine concentrations may have the unintended consequences of increasing toxin exposure to users.

In conclusion, using puffing topography and device setting data from a real-world sample of e-cigarette users, use of a lower (6 mg/mL) nicotine concentration e-liquid was associated with higher formaldehyde and acetaldehyde exposure regardless of whether voltage settings were fixed or adjustable, resulting in an estimated 2 fold increase in cancer risk. Our results also suggest that the compensatory behavior is not sufficient to compensate nicotine mouth-exposure. A combination of effective nicotine delivery (to reduce compensatory puffing) and low carbonyl emissions may reduce potential health risk from vaping. As changing puffing behaviors might be unconscious, vapers should exercise caution when transitioning to lower nicotine concentrations.

## Methods

### Human puffing topography data

Nineteen participants took part in the study as previously described^31,47^. Ethical approval (including all experimental protocols) was granted by London South Bank University (Application reference: UREC 1604) and has been funded by Cancer Research UK (Application reference: C50878/A21130). Informed consent was collected in writing at the baseline session at the University prior to any data collection. A statement to confirm that all methods were carried out in accordance with relevant guidelines and regulations. All methods were carried out in accordance with relevant guidelines and regulations

Participants used an eVic Supreme™ by Joytech fitted with a ‘Nautilus Aspire’ 5 mL tank housing a BVC atomizer (1.6ohm) for at least one week in each of four counterbalanced conditions. Conditions included, 6 mg/mL nicotine with a fixed 4.0 V battery output voltage (6 Fixed [6F]); 18 mg/mL nicotine with a fixed 4.0 V battery output voltage (18 Fixed [18F]); 6 mg/mL nicotine with adjustable (as desired by the user) output voltage (6 Adjustable [6 A]); and 18 mg/mL nicotine with adjustable output voltage (18 Adjustable [18 A]). Participants had a choice of four e-liquid (though participants were obliged to use the same flavor through the whole experiment). The e-cigarette recorded details of each puff parameter, including time, puff duration (PD) and battery output voltage (BOV) for 6 and 18 adjustable (A) settings throughout the 4 weeks. Inter-puff intervals (IPI) were calculated by subtraction between duration of the puff by the time of the previous puff. Puff duration and battery output voltage were characterized with a normal distribution, so for each participant in each condition (6F, 18F, 6A, 18A) mean values were calculated. As the inter-puff interval raw data where highly skewed, the median value was calculated, as the most reasonable description of the central tendency of IPI (See Dawkins *et. al*.^31^). Due to device error, puffing topography data was collected for 19/20 participants.

### E-liquids

Participants chose from 4 different e-liquid flavors (tobacco, fruit, bakery and menthol), and were required to use the same e-liquid for the duration of the study (in both 6 and 18 mg/mL nicotine conditions). The same e-liquid used by each participant was used in the laboratory to mimic puffing behaviors. In total, 7 e-liquids (3 fruit flavors, 2 menthol flavors, one tobacco and one sweet/bakery) were chosen from two different manufacturers. E-liquids were analyzed for nicotine and carbonyl compound using the methods described below. For analysis, 50 µL of e-liquid was added to sorbent removed from sorbent tubes (carbonyl compounds) and stored in the refrigerator for 24 h, or diluted in 10 mL of methanol (nicotine). To measure density of e-liquids, 50 µL of e-liquid was weighted and density was calculated dividing the mass/volume. Three bottles of each e-liquid were tested each time.

Density of e-liquids varied from 1.14 to 1.17 g/mL with a mean of 1.16 ± 0.01 g/mL. Results of the nicotine and carbonyl content analysis are presented in Supplementary Table 1. Mean concentration of nicotine was 5.59 ± 0.75 and 18.03 ± 1.24 respectively for 6 mg/mL and 18 mg/mL e-liquids. The highest difference between label and actual content was 30% (e-liquid e: 6 mg/mL, fruit flavor).

Formaldehyde was detected in 6/7 e-liquids (in both the 6 mg/mL and 18 mg/mL nicotine concentrations), with concentrations varying from 1.11 ± 0.10 (e-liquid b: menthol flavor) to 4.66 ± 0.67 µg/mL (e-liquid g: blueberry muffin flavor). The mean value of formaldehyde was 2.0 ± 1 µg/mL (2.40 ± 1.24 and 1.58 ± 0.38 for 6 and 18 mg/mL e-liquids respectively). The highest concentration of formaldehyde was found in the bakery flavor (g) 6 mg/mL e-liquid: 4.66 ± 0.67 vs 1.85 ± 0.11 µg/mL for 18 mg/mL (p < 0.05). Two out of three fruit flavors also had significantly higher levels of formaldehyde in the 6 mg/mL vs the 18 mg/mL nicotine concentrations (2.79 ± 0.26 vs 1.22 ± 0.12, and 2.59 ± 0.08 vs 1.51 ± 0.17 µg/mL for 6 vs 18 mg/mL nicotine concentrations respectively.

Acetaldehyde was detected in all e-liquids in concentrations varying from 0.84 ± 0.01 (18 mg/mL e-liquid b: menthol flavor) to 293.18 ± 13.68 µg/mL (6 mg/mL e-liquid g: bakery flavor). The mean concentration for acetaldehyde was 54.89 ± 96.24 µg/mL (55.64 ± 103.87 and 54.15 ± 90.55 for 6 and 18 mg/mL e-liquids respectively). One brand (e-liquid c: menthol flavor) had statistically lower concentration of acetaldehyde in 6 compared with 18 mg/mL e-liquid (0.95 ± 0.02 vs 34.97 ± 1.13 µg/mL) and one had higher in 6 compared with 18 mg/mL (e-liquid g: ‘Captain’s curse’ blueberry muffin flavor): 293.18 ± 13.68 vs 260.76 ± 4.42 µg/mL).

### E-cigarette preparation and aerosol generation

The same e-cigarette and tank model (described above) used by participants in the Dawkins *et al*.^31^ study was used to mimic puffing patterns in the laboratory. Separate tanks were used for each e-liquid strength and brand. The device was set up with the widest air-flow and the mean battery output voltage used by each participant during each condition. Tanks were filled with 4 mL of e-liquid (80% of capacity) one day before the experiment. Tanks were rolled for one minute to ensure the wick was soaked and to distribute e-liquid uniformly in the tanks in the time of experiment. Tanks were stored in the dark between experiments. E-cigarettes were charged the day before the experiment and swapped after 15 puffs (one series of aerosol generation) if the level of battery capacity dropped below 50%.

Aerosol from e-cigarettes were generated using the automatic smoking machine Palaczbot (University of Technology, Lodz, Poland) as described previously^22,30^. In the current study we used PD, IPI and BOV obtained for each participant recorded by their device through the four-week period in each condition with the e-liquid and device used to test for nicotine, aerosol yield and carbonyls in laboratory condition. Puff volume was fixed to 70 mL.

Amount of vaporized e-liquid was measured by subtracting the mass of the tank after aerosol generation from the mass of the tank before aerosol generation for nicotine analysis as describing previously^23^.

Aerosol yield was generated 3 times for each analysis (for nicotine or carbonyl compounds) to replicate each participant’s puffing patterns. Aerosol was tested for carbonyl compounds (i.e. formaldehyde, acetaldehyde, acrolein, benzaldehyde) and nicotine emission with puffing regimes.

### Chemical analysis

The method of aldehydes and ketones determination involves an adsorption of aldehydes and ketones aerosol mixture on a pipe filled with silica gel saturated with 2,4-dinitrophenylhydrazine, desorption of the compounds with acetonitrile in an ultrasound washer, and determination using reversed phase high performance liquid chromatography on an AT 1200 liquid chromatograph (Agilent Technologies) equipped with a Zorbax Eclipse PAH column (4,6 ×250 mm, 5μm) and spectrophotometric detector DAD with detection at 360 nm. This allows determination of the following compounds: formaldehyde, acetaldehyde, acrolein, acetone, propionic aldehyde, crotonaldehyde, butanal, benzaldehyde, isovaleric aldehyde, valeric aldehyde, m-methylbenzaldehyde, o-methylbenzaldehyde, p-methylbenzaldehyde, hexanal, 2,5-dimethylbenzaldehyde^3,23^. Limits of detection per sample (50 µL of e-liquid) and per puff are presented in Supplementary Table 2.

Nicotine captured on Cambridge filter was desorbed using 10 mL of methanol with quinoline as an internal standard, and was analysed using gas chromatography with Thermionic Specific Detector (GC-TSD, Varian Inc.)^21,22,47^.

### Statistical analysis

Daily exposure was calculated by multiplying the aerosol, nicotine or carbonyl yield per puff by the mean daily number of puffs taken by each participant in each condition.

Nicotine and carbonyl levels in e-liquids were analyzed using repeated measures analysis of variance (ANOVA). In cases where levels were non quantifiable (ND, BLQ), the median is presented in the results table.

For daily exposure analysis, due to the highly skewed carbonyl data, medians for each replication for each participant under each condition (6F, 18F, 6A, 18A) are presented. Nicotine and aerosol daily exposure was normally distributed but for sake of consistency, we report medians and nonparametric analysis throughout although means and SDs are also presented for all variables in Table 1 for sake of completeness.

In cases where a compound was not detected, 0 was used for statistical analysis. When data were below level of detection, the most conservative approach was adopted by taking the middle value of the range between the limit of quantification and detection for analysis (LD + (LQ-LD)/2).

Non-parametric statistical tests for repeated measures ordinal data (Friedman test) were conducted to compare the four condition (6F, 18F, 6A, 18A) (3 degrees of freedom) to detect whether the number of people for whom an increase or decrease was shown between conditions was statistically significant for aerosol yield, nicotine, formaldehyde and acetaldehyde (as potential carcinogens). Where significant, post-hoc Wilcoxon signed-rank tests (two-sided) for repeated measurements was conducted (for 6F vs. 6 A; 18 F vs. 18 A; 6 F vs. 18 F and 6 A vs. 18 A). The accepted alpha level was 0.05.

Statistical analysis was performed using Statistica and IBM SPSS. Raw data including basic statistics (means, medians and checking for normal distribution) were analyzed using Statistica 13.3 (TIBCO Software Inc.). The same software was used to analyze levels of nicotine and carbonyls in e-liquids. The remaining analysis was performed using IBM SPSS Statistics 24^th^ release (IBM Corp.).

### Changes in cancer risk potencies

The inhalation unit risk (U) for carcinogen is defined as the excess lifetime cancer risk from continuous inhalation exposure to 1 µg of the carcinogen per m3.^48^ Thus, the cancer risk indices (CRI) for each compound might be calculated by dividing daily exposure in µg/day by the default breathing rate of 20 m3/day and multiplying by unit risk (µg/m^3^)^−1^ ^49^. Although in our study only 2 carcinogens were quantified (formaldehyde – IARC 1 type and acetaldehyde – IARC 2b type), they represent more than 95% of cancer potency in e-cigarette aerosol according to Stephens^2^. The Office of Environmental Health Hazard Assessment unit risk values were used as previously^2^. U, according to this database, is 6.0 × 10^−06^ and 2.7 × 10^−06^ for formaldehyde and acetaldehyde respectively. To compare cancer potency from using e-cigarettes with different settings we calculated change factors by dividing the sum of cancer risk indices from the 18 mg/mL nicotine e-liquid use by 6 mg/mL nicotine use in both F and A settings for median values. Calculated CRIs were not compared statistically, as they are calculated from already analyzed data by multiplying/dividing results by constant values.

## Supplementary information

Supplementary Dataset 1.
