# Supplementary material for: Daily exposure to formaldehyde and acetaldehyde and potential health risk associated with use of high and low nicotine e-liquid concentrations
Source: Sci Rep. 2020 Apr 16;10:6546. doi: 10.1038/s41598-020-63292-1 (PMC7162853; doi:10.1038/s41598-020-63292-1)
Supplement: Supplementary file 1 — Supplementary Dataset 1. [file 41598_2020_63292_MOESM1_ESM.docx]

**Title: Daily exposure to formaldehyde and acetaldehyde and potential health risk associated with use of high and low nicotine e-liquid concentrations**

Kosmider Leon^1*^, Cox Sharon^2^, Zaciera Marzena^3^, Kurek Jolanta^3^, Goniewicz Maciej L.^4^, McRobbie Hayden^5^, Kimber Catherine^2^, Dawkins Lynne^2^

^1^ Department of General and Inorganic Chemistry, School of Pharmacy with the Division of Laboratory Medicine in Sosnowiec, Medical University of Silesia in Katowice, 41-200 Sosnowiec, VA 23298, Poland.

^2^ Centre for Addictive Behaviours Research, School of Applied Sciences, London South Bank University, SE1 0AA, London, UK

^3^ Department of Chemical Hazard and Genetic Toxicology, Institute of Occupational Medicine and Environmental Health, 41-200, Sosnowiec, Poland

^4^ Roswell Park Cancer Institute, Department of Health Behavior, Buffalo, NY 14263, USA

^5^ Queen Mary University of London, Wolfson Institute of Preventive Medicine, Barts and The London School of Medicine and Dentistry, E1 4NS, London, UK

**Corresponding author:** Leon Kosmider, Department of General and Inorganic Chemistry, School of Pharmacy with the Division of Laboratory Medicine in Sosnowiec, Medical University of Silesia in Katowice, Jagiellonska 4, 41-200 Sosnowiec, Poland; email: leon.kosmider@gmail.com

Supplementary table 1. Results of e-liquid analysis for nicotine and carbonyls (each e-liquid was analyzed 3 times from 3 different bottles)

| **Code** | **nicotine mean ± SD [mg/ml]** | | **formaldehyde [µg/ml]** | | **acetaldehyde [µg/ml]** | | **acrolein [µg/ml]** | | **acetone [µg/ml]** | | **propionaldehyde [µg/ml]** | | **crotonaldehyde [µg/ml]** | | **butyraldehyde [µg/ml]** | | **benzaldehyde [µg/ml]** | | **isovaleraldehyde [µg/ml]** | | **valeraldehyde [µg/ml]** | | **o-methylbenzaldehyde [µg/ml]** | | **m- methylbenzaldehyde [µg/ml]** | | **p- methylbenzaldehyde [µg/ml]** | | **hexanal [µg/ml]** | | **2,5-dimetylobenzalde [µg/ml]** | |
| --- | --- | --- | --- | --- | --- | --- | --- | --- | --- | --- | --- | --- | --- | --- | --- | --- | --- | --- | --- | --- | --- | --- | --- | --- | --- | --- | --- | --- | --- | --- | --- | --- |
|  | 6 mg/mL | 18 mg/mL | 6 mg/mL | 18 mg/mL | 6 mg/mL | 18 mg/mL | 6 mg/mL | 18 mg/mL | 6 mg/mL | 18 mg/mL | 6 mg/mL | 18 mg/mL | 6 mg/mL | 18 mg/mL | 6 mg/mL | 18 mg/mL | 6 mg/mL | 18 mg/mL | 6 mg/mL | 18 mg/mL | 6 mg/mL | 18 mg/mL | 6 mg/mL | 18 mg/mL | 6 mg/mL | 18 mg/mL | 6 mg/mL | 18 mg/mL | 6 mg/mL | 18 mg/mL | 6 mg/mL | 18 mg/mL |
| a | 6.20 ± 0.03 | 17.12 ± 0.50 | ND | ND | 86.53 ± 1.08 | 77.09 ± 0.34 | 6.04 ± 0.30 | 5.55 ± 0.10 | ND | ND | ND | ND | 2.87 ± 0.60 | 2.42 ± 0.22 | ND | ND | ND | ND | ND | ND | ND | ND | ND | ND | ND | ND | 1.91 ± 0.13 | 2.60 ± 0.08 | ND | ND | BLQ | BLQ |
| b | 6.16 ± 0.27 | 16.91 ± 0.44 | 1.11 ± 0.10 | 1.54 ± 0.31 | 0.98 ± 0.02 | 0.84 ± 0.01 | ND | ND | ND | ND (N=2); | ND | ND | ND | ND | BLQ | BLQ | ND | BLQ (N=2); | ND | ND | BLQ | BLQ | ND | ND | ND | ND | 1.28 ± 0.07 | BLQ | BLQ | BLQ | ND | ND |
| c | 5.29 ± 0.05 | 18.47 ± 0.822 | 1.85 ± 0.27 | 1.18 ± 0.02 | 0.95 ± 0.02 | 34.97 ± 1.13 | ND | ND | BLQ | 3.47 ± 0.55 | ND | BLQ | ND | ND | ND | ND | ND | ND | ND | ND | ND | ND | ND | ND | BLQ | BLQ | 3.56 ± 0.58 | 2.73 ± 0.10 | 0.87 ± 0.04 | 0.57 ± 0.06 | BLQ | BLQ |
| d | 5.01 ± 0.04 | 19.08 ± 0.08 | 1.42 ± 0.45 | 2.19 ± 0.12 | 1.47 ± 0.09 | 0.96 ± 0.03 | BLQ | ND | 2.06 ± 1.56 | 4.91 ± 0.32 | BLQ | BLQ | BLQ (N=2) | BLQ | ND | BLQ | BLQ | 0.49 ± 0.06 | ND | ND | 1.40 ± 0.12 | BLQ | ND | ND | BLQ | 1.47 ± 0.08 | 1.46 ± 0.07 | 1.85 ± 0.11 | 0.65 ± 0.06 | BLQ | 1.11 ± 0.08 | BLQ |
| e | 6.01 ± 0.18 | 19.68 ± 0.35 | 2.79 ± 0.26 | 1.22 ± 0.12 | 3.38 ± 0.19 | 2.52 ± 0.02 | 93.40 ± 1.88 | 81.34 ± 2.63 | 1.47 ± 0.17 | 1.74 ± 0.10 | ND | ND | ND | ND | BLQ | 0.65 ± 0.06 | 3.94 ±0.04 | 2.80 ± 0.32 | BLQ | BLQ | 0.84 ± 0.08 | ND | ND | ND | ND | ND | 3.96 ± 0.34 | BLQ | 187.77 ± 8.89 | 179.62 ± 46.57 | ND | ND |
| f | 4.21 ± 0.20 | 17.52 ± 1.84 | 2.59 ± 0.08 | 1.51 ± 0.17 | 2.96 ± 0.02 | 1.93 ± 0.27 | 1.58 ± 0.09 | 1.85 ± 0.04 | BLQ (N=2) | BLQ | 1.17 ± 0.09 | 0.99 ± 0.14 | ND | ND | ND | ND (N=2) | 10.01 ± 0.40 | 8.74 ± 0.25 | ND | BLQ | ND | ND | ND | ND | ND | ND | 4.77 ± 0.03 | 3.25 ± 0.15 | ND | ND | ND | ND |
| g | 6.23 ± 0.31 | 17.43 ±0.86 | 4.66 ± 0.67 | 1.85 ± 0.11 | 293.18 ± 13.68 | 260.76 ± 4.42 | 86.86 ± 2.29 | 84.29 ± 0.94 | 2.34 ± 0.61 | 2.51 ± 0.82 | ND | ND | ND | ND | 0.72 ± 0.21 (N=2); | BLQ (N=2) | ND | ND | BLQ (N=1) | BLQ | ND | ND | BLQ | BLQ | ND | ND | 12.47 ± 1.09 | 12.58 ± 0.19 | ND | ND | ND | ND |

Supplementary table 2. Limits of detection (LD) and quantifications (LQ)

|  | **E-Liquid [µg/mL]** | | **Aerosol [ng/puff]** | |
| --- | --- | --- | --- | --- |
|  | **LD** | **LQ** | **LD** | **LQ** |
| formaldehyde | 0.16 | 0.50 | 0.53 | 1.67 |
| acetaldehyde | 0.16 | 0.50 | 0.53 | 1.67 |
| acrolein | 0.34 | 1.00 | 1.13 | 3.33 |
| acetone | 0.34 | 1.00 | 1.13 | 3.33 |
| propionaldehyde | 0.2 | 0.60 | 0.67 | 2.00 |
| crotonaldehyde | 0.16 | 0.50 | 0.53 | 1.67 |
| butyraldehyde | 0.16 | 0.50 | 0.53 | 1.67 |
| benzaldehyde | 0.16 | 0.50 | 0.53 | 1.67 |
| isovaleraldehyde | 0.16 | 0.50 | 0.53 | 1.67 |
| valeraldehyde | 0.16 | 0.50 | 0.53 | 1.67 |
| o-methylbenzaldehyde | 0.46 | 1.40 | 1.53 | 4.67 |
| m- methylbenzaldehyde | 0.46 | 1.40 | 1.53 | 4.67 |
| p- methylbenzaldehyde | 0.34 | 1.00 | 1.13 | 3.33 |
| hexanal | 0.16 | 0.50 | 0.53 | 1.67 |
| 2,5-dimetylobenzalde | 0.34 | 1.00 | 1.13 | 3.33 |
